# Improvement of oxidative stress status by lipoprotein apheresis in Chinese patients with familial hypercholesterolemia

**DOI:** 10.1002/jcla.23161

**Published:** 2019-12-20

**Authors:** Jun Wen, Qian Dong, Geng Liu, Ying Gao, Xiao‐Lin Li, Jing‐Lu Jin, Jian‐Jun Li, Yuan‐Lin Guo

**Affiliations:** ^1^ Fuwai Hospital National Center for Cardiovascular Disease Chinese Academy of Medical Sciences Peking Union Medical College Beijing China

**Keywords:** familial hypercholesterolemia, lipoprotein apheresis, oxidative stress

## Abstract

**Background and aims:**

Familial hypercholesterolemia (FH) characterized by severe high blood cholesterol levels usually presents an imbalance of systemic oxidative stress (OS). Lipoprotein apheresis (LA), which is the most effective therapy to reduce cholesterol levels, remains unclear in altering OS and scarce in Chinese patient studies. Our study aims to assess the impact of LA on OS status in Chinese patients with FH.

**Methods:**

About 31 patients (22 males, age: 12‐69 years) with FH and receiving LA treatment were consecutive enrolled. Free oxygen radicals test (FORT) and free oxygen radicals defense (FORD) values were determined using the free oxygen radical monitor and kit immediately before and after LA, while blood samples were collected to measure plasma lipid levels and hs‐CRP by conventional methods. Data were analyzed by paired *t* test or rank sum test and Spearman‐rho correlation analysis.

**Results:**

Besides plasma lipid levels, the OS status showed that FORTs were significantly decreased and FORD values significantly enhanced immediately after LA treatment compared with before (both *P* *<* .01). In addition, the correlation analysis showed that the removal rates (△%) of TC were positively related to the increased rates (△%) of FORD value (*ρ* = 0.513, *P* = .003); LDL‐C to FORD (*ρ* = 0.39, *P* = .03); Lp(a) to FORD (*ρ* = 0.473, *P* = .007); and non‐HDL‐C to FORD (*ρ* = 0.46, *P* = .009). However, no significant difference in hsCRP was found.

**Conclusions:**

The present study indicated, besides effectively lowering plasma lipid levels, LA could significantly improve OS status in Chinese patients with FH.

**Abbreviations**
ACEI: angiotensin‐converting enzyme inhibition
ApoA1: apolipoprotein A1
ApoB: apolipoprotein B
ARB: angiotensin receptor blocker
BMI: body mass index
CAD: coronary artery disease
CVD: cardiovascular disease
DFPP: double‐filtration plasmapheresis
DLCN: Dutch Lipid Clinic Network
EF: ejection fraction
FH: familial hypercholesterolemia
FORD: free oxygen radicals defense
FORT: free oxygen radicals test
HbA1C: hemoglobin AIc
HDL‐C: high‐density lipoprotein cholesterol
hs‐CRP: high‐sensitivity C‐reactive protein
IG: immunoglobulin
LA: lipoprotein apheresis
LDL‐C: low‐density lipoprotein cholesterol
Lp(a): lipoprotein (a)
non‐HDL‐C: non‐high‐density lipoprotein cholesterol
NT‐proBNP: N‐terminal pro‐brain natriuretic peptide
OS: oxidative stress
ROS: reactive oxygen species
TC: total cholesterol
TG: Triglycerides

**Highlights**

- LA treatment is a safe and effect option for Chinese FH patients in removing atherosclerotic lipoproteins, especially TC, LDL‐C, and Lp(a).
- A single LA treatment had a significant impact on immunoglobulin (especially IgM) in FH patients, but not on hs‐CRP.
- LA treatment has been shown to improve oxidative stress status of Chinese FH patients acutely by decreasing FORTs and enhancing FORD values.
- Positive correlations were shown between percent of changing in major atherosclerotic lipoproteins (TC, LDL‐C, Lp(a), and non‐HDL‐C) and FORD, indicating a complementary effect of LA treatment for anti‐atherosclerosis and amelioration of prognosis in patients with FH.

## 1 INTRODUCTION

Familial hypercholesterolemia (FH) characterized by the anomalous metabolism of low‐density lipoprotein and severe hypercholesterolemia is usually a common autosomal dominant disease.^1^ Patients with FH present increased susceptibility to atherosclerosis and premature cardiovascular disease (CVD). FH patients are recommended to receive lipoprotein apheresis (LA) treatment, when the maximum tolerated lipid‐lowering pharmacotherapy is not effective in achieving an optimal target of serum LDL cholesterol (LDL‐C) concentration. Moreover, there were plenty of researches showing that elevated oxidative stress (OS) in patients with FH enhanced the proatherogenic effect of LDL‐C and participated in the acceleration of atherosclerosis.^2^, ^3^ Oxidative stress is known as an imbalance in increased reactive oxygen species (ROS) production and/or decreased antioxidant defense systems.^4^ In addition, abnormal OS contributes to metabolic disorders and carcinogenesis. Therefore, improvement of OS status may be prone to alleviate atherogenesis as well as diabetes mellitus and cancer.^5^, ^6^, ^7^

Lipoprotein apheresis as the most effective treatment for FH patients is a method to selectively remove lipoproteins containing ApoB components from patients' plasma. LA cannot just reduce high LDL‐C concentration and/or Lp(a) levels, but also effectively alleviate clinical symptoms and signs of FH patients.^8^ In addition, LA treatment is recognized as safe and can improve the prognosis of patients with progressive CVD.^9^, ^10^, ^11^ Besides effectively lowering plasma lipid levels, investigations of a single LA treatment on OS parameters, and on the relationship between blood lipids and these parameters were rare and remained conflicts.^12^, ^13^, ^14^, ^15^, ^16^, ^17^

Hence, our study using a systematic FORT and FORD values aims to assess the impact of lipoprotein apheresis on oxidative stress status of Chinese patients with FH, and to study the relationship between the removal rates of blood lipids and the change rates of oxidative stress.

## 2 MATERIALS AND METHODS

### 2.1 Ethics statement

The study was approved by the Ethical Committee of Fuwai Hospital CAMS ＆ PUMC (2018‐1031), and written informed consents were obtained from all participants in Fuwai Hospital.

### 2.2 Patients and lipoprotein apheresis procedures

Our study included a total of 31 FH patients (22 males, 9 females, age: 12‐69 years) receiving lipoprotein apheresis treatment. These patients were enrolled in our lipid ward from November 2017 to February 2019. The mean body mass index (BMI) was calculated as 25.5 ± 4.6, range 13.0‐34.7, median 24.8 kg/m^2^. The diagnosis of FH patients was in accord with Dutch Lipid Clinic Network (DLCN) criteria.^18^ All patients received statin treatment, and 29 of all patients were identified as CAD by coronary angiography. Inclusion criteria in our study were patients without targeted plasma lipid levels or with high risk of coronary heart disease under lifestyle interventions and maximum tolerated lipid‐lowering medications. Patients with complications of severe heart failure or arrhythmia, systemic acute inflammatory disease, coagulation disorder, and liver dysfunction were excluded. The study also failed to include patients who disagreed to receive the procedure because of the high costs of LA treatment.

Lipoprotein apheresis device of our study was double‐filtration plasmapheresis (Asahi, Kasei Medical), in which a Plasmaflo OP‐08 W membrane‐type plasma separator was used for the primary membrane and a cascadeflo^TM^ EC‐50 W membrane‐type plasma component separator was used for the secondary membrane separation. The whole blood obtained from median cubital or femoral vein was fully heparinized, and the blood flow was 60‐120 mL/min, and the separation flow was 25%‐30% for separation. The target plasma volume was calculated from 2 to 4.8 L based on the patient's body weight and the ratio of red blood cells. Each lipoprotein apheresis treatment lasted around 1‐4 hours, according to the individual's condition. Monitoring of blood pressure and other complications were also substantial throughout the lipoprotein apheresis procedure.

### 2.3 Clinical data

A detailed medical history and physical examination of all participants were obtained at the time of admission. There were two most important time points to collect blood samples, immediately before and immediately after procedure.

### 2.4 Laboratory data

Free oxygen radicals test (FORT) and free oxygen radicals defense (FORD) values were determined using the free oxygen radical monitor and kit (Catellani Group) immediately before and after lipoprotein apheresis, and FORT and FORD assays were previously described.^19^ In addition, blood samples were also collected to measure plasma lipid levels, high‐sensitivity C‐reactive protein (hsCRP), and immunoglobulins (enzymatic assay and turbidimetric immunoassay). The removal rates (△%) of FORT and blood lipids, or the increased rates (△%) of FORD were calculated using the following formula: (pre‐apheresis concentration ‐ post‐apheresis concentration)/pre‐apheresis concentration or (post‐apheresis concentration ‐ pre‐apheresis concentration)/pre‐apheresis concentration.

### 2.5 Statistical analysis

The values were expressed as mean ± SD for normally distributed continuous variables or median (25th‐75th percentile) for those not normally distributed; while the values were expressed as number (percentage) for categorical variables. Paired *t* test or rank sum test were used to analyze the effects for oxidative stress status, plasma lipids, and other blood indexes before and after lipoprotein apheresis treatment. The correlation coefficient between LA treatment‐induced alterations in oxidative stress biomarkers and lipid parameters was obtained by a non‐parametric Spearman‐rho correlation analysis. SPSS statistics software version 25 was used for statistical analysis (SPSS, Chicago, IL, USA). The statistical significance of *P* values indicating two‐tailed significance was considered to be .05 or less.

## 3 RESULTS

Baseline data of patients were shown in the Table 1. Out of 31 FH patients (22 males, 9 females, age: 12‐69 years) receiving lipoprotein apheresis treatment, 16 were smokers and 7 were alcohol users. Beyond that, 14 were diagnosed with hypertension, 5 with diabetes mellitus, and 29 with CAD by coronary angiography, while all patients received statin or statin with ezetimide treatment, 24 received antiplatelet therapy, and 12 received ACEI/ARB treatment.

**Table 1:** Baseline characteristics of study patients

variables	FH (n = 31)
Male/female	22/9
Age, years	41 ± 13
BMI, kg/(m^2^)	25.51 ± 4.55
Smoke, n (%)	16 (52%)
Drink, n (%)	7 (23%)
Hypertension, n (%)	14 (45%)
Diabetes mellitus, n (%)	5 (16%)
CAD, n (%)	29 (94%)
Statin, n (%)	31 (100%)
Antiplatelet therapy, n (%)	24 (77%)
ACEI/ARB, n (%)	12 (39%)
EF, %	62 ± 11
NT‐proBNP, pg/mL	68.05 (28.70, 145.88)
HbA1C, %	6.0 ± 0.9
hsCRP, mg/L	2.00 (1.23, 3.68)

Note

Data are expressed as mean ± SD, median (25th–75th percentile), or n (%).

Abbreviations: ACEI, angiotensin‐converting enzyme inhibition; ARB, angiotensin receptor blocker; BMI, body mass index; CAD, coronary artery disease; EF, ejection fraction; HbA1C, hemoglobin AIc; hsCRP, hypersensitive C‐reactive protein; NT‐proBNP, N‐terminal pro‐brain natriuretic peptide.

### 3.1 Improvement of oxidative stress status

**Figure 1:**
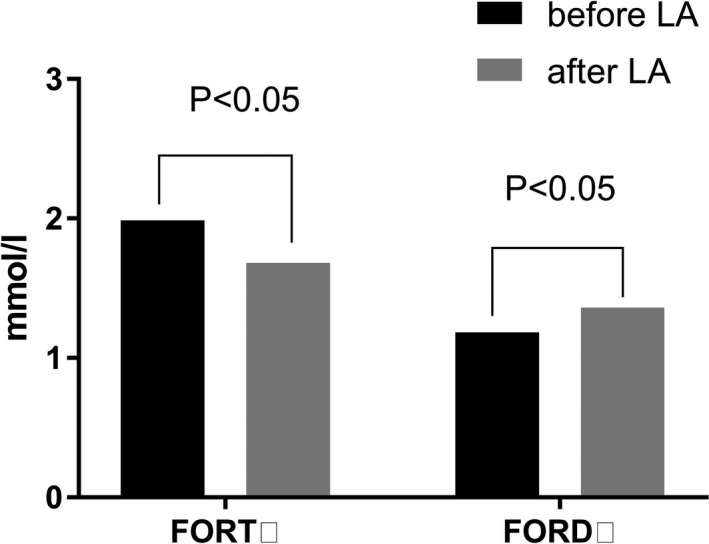
The immediate effects of LA treatment in oxidative stress status. FORT, free oxygen radicals test, FORD, free oxygen radicals defense, LA, lipoprotein apheresis

As can be seen from Figure 1, LA treatment ameliorated the oxidative stress status of FH patients. Our present study showed that FORTs were significantly decreased (13%), and FORD values enhanced (24%) immediately after LA treatment.

### 3.2 Changes in plasma lipids and other components

**Figure 2:**
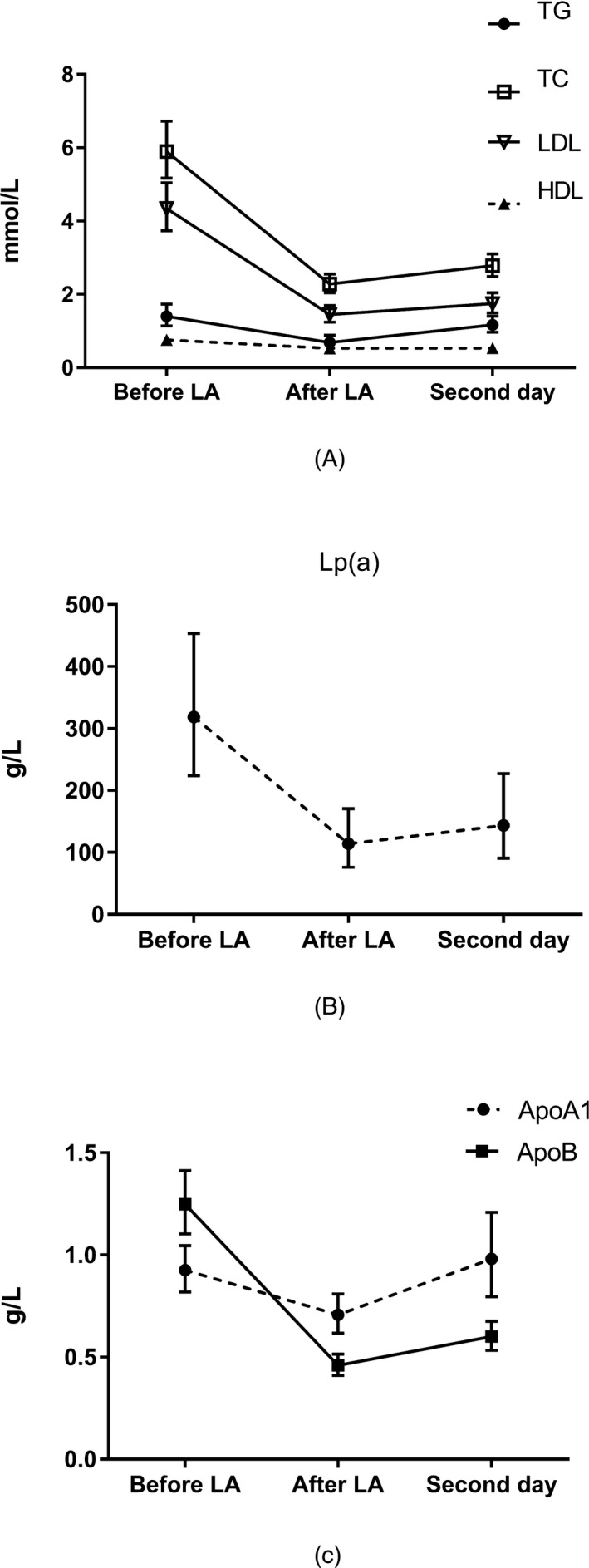
Curve of plasma lipid levels in patients before and after LA treatment. ApoA1, apolipoprotein A1, ApoB, apolipoprotein B, HDL‐C, high‐density lipoprotein cholesterol, LDL‐C, low‐density lipoprotein cholesterol, Lp(a), lipoprotein (a), TC, Total cholesterol, TG, Triglycerides

In Figure 2 and Table 2, LA treatment reduced plasma lipid levels immediately and significantly. The data presented a significant reduction immediately after LA treatment in total cholesterol by 65%, triglyceride by 52%, low‐density lipoprotein cholesterol by 67%, high‐density lipoprotein cholesterol by 31%, lipoprotein (a) by 68%, apolipoprotein A1 by 23%, and apolipoprotein B by 65%. Simultaneously, our study found that LA treatment also had an effect on immunoglobulins, especially on Immunoglobulin M. IgA, G, and M were reduced by 14%, 20%, and 44%, respectively, all *P* < .05. However, the concentrations of hsCRP were not influenced after LA treatment (*P* = .428).

**Table 2:** Comparison of alterations in plasma lipid levels before and after LA treatment

Time Points	TG	TC	LDL‐C	HDL‐C	Lp(a)	ApoA1	ApoB
	mmol/L	mmol/L	mmol/L	mmol/L	mg/L	g/L	g/L
Before	1.25 (0.98, 1.85)	6.10 (4.12, 7.55)	4.46 (2.91, 5.64)	0.81 ± 0.27	338.0 (142.0, 699.6)	0.97 ± 0.28	1.25 (0.94, 1.55)
After	0.60 (0.39, 1.22)**	2.16 (1.87, 2.79)**	1.40 (1.12, 1.84)**	0.56 ± 0.20**	108.6 (50.1, 226.0)**	0.75 ± 0.22**	0.44 (0.38, 0.56)**
Second day	1.21 (0.79, 1.62)*	2.74 (2.17, 3.45)**	1.67 (1.33, 2.16)**	0.57 ± 0.20**	140.7 (70.7, 368.9)**	0.96 (0.73, 1.06)	0.58 (0.50, 0.71)**

Abbreviations: ApoA1, apolipoprotein A1; ApoB, apolipoprotein B; HDL‐C, high‐density lipoprotein cholesterol; LDL‐C, low‐density lipoprotein cholesterol; Lp(a), lipoprotein (a); TC, Total cholesterol; TG, Triglycerides.

* Statistically significant (*P* < .05) with paired *t* test or rank sum test.

** Statistically significant (*P* < .01) with paired *t* test or rank sum test.

### 3.3 Correlation analysis

Significant alterations in both lipid parameters and oxidative stress biomarkers were observed in our study. In Spearman‐rho correlation analysis, the increased rates (△%) of FORD were well correlated with the removal rates (△%) of TC (*ρ* = 0.509, *P* = .003), LDL‐C (*ρ* = 0.39, *P* = .03), Lp(a) (*ρ* = 0.473, *P* = .007), and non‐HDL‐C (*ρ* = 0.46, *P* = .009). However, no other statistical correlations between lipid parameters and oxidative stress biomarkers were obtained, Table 3.

**Table 3:** Correlation between the rates of change (△%) in lipid parameters and oxidative stress biomarkers after LA treatment

Lipid parameters (△%)	FORT (△%)	FORD (△%)
TG	−0.133	0.077
	NS	NS
TC	0.121	**0.509**
	NS	***P* = .003**
LDL‐C	0.213	**0.390**
	NS	***P* = .03**
HDL‐C	0.171	0.136
	NS	NS
Lp(a)	0.238	**0.473**
	NS	***P* = .007**
Non‐HDL‐C	0.069	**0.460**
	NS	***P* = .009**
Remnant cholesterol	−0.145	0.080
	NS	NS

Note

*P* indicates two‐tailed significance with Spearman‐rho correlation analysis. Bold indicates statistical significant value (*P* < .05).

Abbreviations: FORD, free oxygen radicals defense; FORT, Free oxygen radicals test; non‐HDL‐C, Non‐high‐density lipoprotein cholesterol; NS, not significant.

## 4 DISCUSSION

Lipoprotein apheresis treatment is a safe and effective option for FH patients who have received maximum tolerated lipid‐lowering pharmacotherapy and whose LDL‐C concentration is still above the target levels. In our study, LA treatment not only reduced plasma lipid levels of Chinese patients with FH, but also improved oxidative stress status by reducing FORT 13% and increasing FORD 24%. Moreover, the correlations in the removal rates of lipid parameters and the increased rates of antioxidant biomarkers indicated complementary effect of LA.

The LA equipment used in our study was double‐filtration plasmapheresis (DFPP). Compared with the selective purification device, DFPP has similar effects of lowering LDL‐C and Lp(a) concentrations, but it also has a scavenging effect on HDL‐C and TG concentrations.^20^ In present study, the effects of DFPP on TC, TG, LDL‐C, HDL‐C, and LP (a) were 65%, 52%, 67%, 31%, and 68%, respectively, which was similar to the results reported by Albayrak et al^21^ previously. In addition, compared with the results of the next day, the plasma lipid levels rebounded immediately after the end of LA treatment, because lipid metabolism has been ongoing and the genotype of FH patients is correlated with LDL‐C rebound velocity.^22^ Thus, these results suggested that a single LA treatment can immediately remove atherosclerotic cholesterol of FH patients.

Familial hypercholesterolemia is characterized by elevated concentrations of LDL‐C from birth, which can lead to increased susceptibility to atherosclerosis and premature cardiovascular morbidity and mortality.^1^ Furthermore, the oxidative stress status is significantly increased in FH patients.^2^ Our study showed that the oxidative stress status of FH patients were significantly improved by a single LA treatment, with reducing FORT 13% and increasing FORD 24%, which was in agreement with previous study showing that LA treatment can decrease OS biomarkers acutely,^23^ and enhance antioxidant capability.^12^ Oxidative stress is an imbalance between oxidation and antioxidant defense systems and is usually along with chronic inflammatory condition, notably in the pathogenesis of atherosclerosis, metabolic disorders, and cancer.^4^, ^6^, ^7^, ^24^, ^25^ The elevated OS and dysfunctional lipid metabolism enhance inflammation and insulin resistance and also lead to mitochondrial damage and genomic instability, which pave the way for the development of metabolic disorders and carcinogenesis. All of these suggest that alleviating lipid metabolism and OS status may be a feasible means in reducing the incidence of metabolic disorders and cancer. In present study, a single LA treatment reduced both blood lipid levels and FORT values and increased FORD values. Additionally, the correlation analysis suggested that the removal rates of TC (△%) was positively related to the increased rates of FORD value (△%) after LA treatment (*ρ* = 0.513, *P* = .003), LDL‐C to FORD (*ρ* = 0.39, *P* = .03), Lp(a) to FORD (*ρ* = 0.473, *P* = .007), and non‐HDL‐C to FORD (*ρ* = 0.46, *P* = .009), but other lipid parameters had no statistical correlations with oxidative stress biomarkers, Table 3. Similar results were found by Kopprasch et al,^13^ showing that LDL‐C and TC were positively correlated with anti‐oxidized LDL antibodies, rather than Lp(a). These results, hence, showed that LA treatment reduced blood lipid levels and improved oxidative stress status, which indicated LA treatment may be not just useful in the prevention of CVD, but also play a role in metabolic disorders and even carcinogenesis in patients with FH.

Cellular dysfunction is major pathological manifestation in cancer development, and cancer cells are characterized by increased aerobic digestion (known as the Warburg effect) and high levels of oxidative stress.^26^ Increased oxidative stress along with chronic inflammatory has been linked with DNA damage and subsequent malignancies.^27^ Previous study showed that increased OS, and decreased antioxidant capacity and HDL‐C concentrations, may impact the pathogenesis of diffuse large B‐cell lymphoma.^25^ Similarly, metabolic disorders with high OS were involved in abnormal cell proliferation, apoptosis, and energy metabolism and were related to carcinogenesis.^6^ The implications for OS regulation and alleviation of metabolic disorders are highly significant for long‐term benefits of cancer occurrence and treatment. Our study reported the effect of a single LA treatment with improving OS status and decreasing blood lipids in FH patients. Therefore, the LA treatment may also play a better role in suppressing carcinogenesis.

Our study also found that immunoglobulins A, G, and M were reduced by 14%, 20%, and 44%, respectively. Julius and coworkers reported similar results that a single LA treatment had an effect on lowering immunoglobulin concentrations, especially IgM.^28^ However, the concentrations of hsCRP were not influenced significantly by LA treatment in our study, which was inconsistent with previous results.^29^, ^30^ In our study, no significant changes in hsCRP may be associated with establishment of a vascular channel that can cause an inflammatory response and the short interval (<24 hours) between two blood sample collections. Besides, whole blood needs to be separated and in contact with artificial pipes during treatment, which may affect the results of hsCRP.

The main limitations in our study included relatively small sample size. The small number of participants in present study may affect the efficacy of our results. Furthermore, since the LA treatment technique is carried out only in few big medical centers and the price of LA treatment is very high without government medical insurance supporting in China currently, many FH patients are unable to receive LA treatment, resulting in a certain bias from patient selection.

## 5 CONCLUSION

The present study indicated, besides effectively lowering plasma lipid levels, a single lipoprotein apheresis treatment could significantly improve oxidative stress status in Chinese patients with FH. This effect of LA treatment might be complementary to the decrease of LDL‐C and inhibit the progress of atherosclerosis, and might even be associated with the prevention of metabolic disorders and carcinogenesis. However, our study only showed the effect of a single immediate LA treatment, continuous oxidative stress monitoring after LA treatment, and long‐term treatment efficacy need to be further clarified.

## CONFLICT OF INTEREST

None declared.

## AUTHOR CONTRIBUTIONS

JW analyzed data and wrote the article; QD and GL measured FORT and FORD values and contributed to data collection; YG and X.‐LL contributed to patients enrolling and clinical diagnosis of disease; J.‐LJ contributed to data management and analysis; J.‐JL designed the study and revised the article; Y.‐LG is the designer of this study, had full access to all the data analysis in the study, and takes responsibility for the writing instruction; All authors have read and approved the final article.
